# The Value of Greenspace Under Pandemic Lockdown

**DOI:** 10.1007/s10640-020-00489-y

**Published:** 2020-08-04

**Authors:** Brett H. Day

**Affiliations:** Land Environment Economics and Policy Institute, Department of Economics, University of Exeter, Exeter, UK

**Keywords:** COVID-19, Google mobility data, Latent class regression, Recreation demand model, Non-market valuation

## Abstract

The COVID-19 outbreak resulted in unprecedented restrictions on citizen’s freedom of movement as governments moved to institute lockdowns designed to reduce the spread of the virus. While most out-of-home leisure activities were prohibited, in England the lockdown rules allowed for restricted use of outdoor greenspace for the purposes of exercise and recreation. In this paper, we use data recorded by Google from location-enabled mobile devices coupled with a detailed recreation demand model to explore the welfare impacts of those constraints on leisure activities. Our analyses reveals evidence of large-scale substitution of leisure time towards recreation in available greenspaces. Indeed, despite the restrictions the economic value of greenspace to the citizens of England fell by only £150 million over lockdown. Examining the outcomes of counterfactual policies we find that the imposition of stricter lockdown rules would have reduced welfare from greenspace by £1.14 billion. In contrast, more relaxed lockdown rules would have delivered an aggregate increase in the economic value of greenspace equal to £1.47 billion.

## Introduction

As the COVID-19 pandemic swept across the planet, national governments instituted various rules designed to reduce human contact and slow rates of infection. The severity of these lockdown rules differed from nation to nation, largely mirroring the severity of the virus outbreak. This paper focuses on England, whose own lockdown experience began on 23rd March, 2020. The lockdown in England placed unprecedented restrictions on citizen’s freedom of movement. As well as not being able to go to their places of work, citizens were deprived of access to most shops, food and drink outlets, entertainment establishments and leisure facilities. One of the few privileges that remained was the opportunity to spend time outdoors walking and exercising, activity often undertaken in greenspace. This paper presents an empirical exploration of the levels of engagement with greenspace over the lockdown in England. It focuses on the question of how greatly the lockdown rules impacted on the value flows realised by English citizens from their greenspace and explores how those impacts might have differed had stricter or more relaxed restrictions been imposed.

A priori, it is not self-evident whether the value derived from greenspace as a focus for outdoor recreation was diminished or amplified by the rules of lockdown and the conditions of the COVID-19 outbreak. On the one hand, citizens may have reduced their use of greenspaces in an effort to minimise their risks of exposure to the virus. Likewise, lockdown rules prevented citizens from visiting all but highly local greenspaces. Limiting citizens’ options to a small set of potentially less-desirable destinations will again have acted to dampen demand. On the other hand, under lockdown, citizens were unable to participate in nearly all other forms of out-of-home leisure activity, demand for greenspace may have increased as citizens substituted away from those unavailable alternative uses of their leisure time. In addition, under lockdown many citizens were unable to work. Releasing the usual-leisure time constraints on those individuals will also have acted to increase demand for outdoor recreation.

As the lockdown unfolded, localised evidence of changing behaviour arose. Newspaper reports described normally busy beaches as all but deserted (Betts 2020; Crane 2020; Ikonen 2020). In contrast, incidents of overcrowding in city greenspaces resulted in temporary closures of several large urban parks (including London’s Brockwell Park and Victoria Park as well as Middlesbrough’s Stewart Park). In this paper we make use of data collected by Google from location-enabled mobile devices which provides systematic evidence on the rates of visitation to greenspace across the regions of England over the course of the lockdown (Google 2020). As described in Sect. 3, this Google Mobility data reveals that demand for greenspace changed over the course of the lockdown in ways which mirror the evolving rules on outdoor activity.

The second key resource used in this paper is the Outdoor Recreation Valuation (ORVal) model (Day and Smith 2017), which we use not only to predict demand for visits to greenspace under the restrictive rules of the lockdown but also to estimate the changes in economic value experienced by residents of England as a consequence of those rules. Developed in partnership with the UK government,^1^ ORVal is underpinned by an econometric model estimated in the random utility framework. As such, ORVal follows in a tradition stretching back at least as far as Kocur et al. (1979) and Feenberg and Mills (1980), What distinguishes ORVal from other such models is that it is, as far as we are aware, the first to consider the entire range of publically-accessible greenspace sites including parks, gardens, playing fields, church yards, cemeteries, allotments, nature reserves, woodlands, wetlands, river and lakeside walks, beaches and the network of coastal and countryside paths. We briefly review the ORVal model in Sect. 4.

Of course, ORVal was estimated on data in which individuals were not concerned with risking exposure to a deadly virus, in which their pursuit of alternative leisure activities was unrestricted and where they faced the leisure-time constraints of normal working conditions. In this paper, we assume that differences between the ORVal predictions of recreation behaviour under the lockdown rules and those observed in the Google mobility data are the net result of those, and possibly other, factors. As described in Sect. 5, we undertake a novel statistical exercise in model calibration using techniques of latent class regression to estimate parameters for the ORVal model which capture the net effect of those factors on recreation behaviour. Those estimates allow us to construct a times series of ORVal predictions for recreation activity under the rules of the lockdown that can be contrasted to a counterfactual in which COVID-19 had not come to pass. We present the findings from that comparison in Sect. 6. In brief, we find that while the lockdown imposed very significant restrictions on outdoor recreation activities, citizens engaged in substantial compensating substitution behaviour. The mitigating effect of that substitution behaviour meant that over the lockdown, citizens of England experienced only a 2.1% fall in the welfare they might otherwise have enjoyed from greenspace, an amount equating to a loss in aggregate economic value of £150 million.

Our calibration of the ORVal model allows us to explore other counterfactuals; namely, how engagement with the outdoors might have proceeded through the COVID-19 outbreak under stricter or under more relaxed lockdown rules. Not surprisingly, we find that in the strict-rules counterfactual welfare from greenspace is £1.14 billion lower than under the actual lockdown rules. In contrast, applying less strict lockdown rules on outdoor recreation allows for even greater use of the outdoors and delivers an aggregate welfare benefit of £1.47 billion.

## Literature Review

This paper’s contribution is primarily empirical. It attempts to quantify the impact of the COVID-19 pandemic and its associated lockdown on one aspect of a nation’s everyday life; outdoor recreation in greenspace. Not surprisingly, given the recency of the events, little exists in the published literature with a similar intent. An unpublished manuscript by Venter et al. (2020) examines changes in outdoor activity in Oslo, Norway during the virus outbreak. Using data on the route choices of runners and cyclists, they find that spatial patterns of exercise activity changed over lockdown to favour greener and more remote locations. Through a calibration exercise, Venter et al. estimate that outdoor recreation activity in Oslo increased by291%. In another yet to be published manuscript, Rice and Pan (2020) explore data made publically available by Google on the use of greenspace during the COVID-19 pandemic, data that we also exploit in our study (Google 2020). Focusing on 111 counties in the western United States, they identify an average 2.5% increase in greenspace visitation and find that differences across counties are chiefly explained through differences in weather.^2^

Our study differs from these other contributions in a number of ways. The focus of our study is England, where lockdown rules on recreation were not dissimilar to those in the western US but significantly stricter than in Oslo.^3^ Rather than routes used for exercise we explore visits to greenspace. And unlike both Venter et al. (2020) and Rice and Pan (2020), our focus is not primarily on how recreation patterns changed over space, but how they responded to changes in lockdown rules. Perhaps the clearest point of separation is that we are the first to attempt to attribute an economic value to the changes in greenspace use that arose over the lockdown.

## Engagement with the Outdoors Over the Lockdown in England

### Timeline of the Lockdown

The English lockdown began on March 23rd, 2020 with non-essential workers asked to work from home. Shops and entertainment outlets were forced to close unless selling essential items and travel was only allowed if absolutely necessary. Our particular interest concerns the rules on outdoor recreation for which specific guidelines were issued People were expected to use open spaces near to their homes and encouraged to limit themselves to one trip a day. Driving to open spaces for the purposes of outdoor recreation was not allowed (HC Deb 24th March 2020). Requirement to abide by these measures was passed into law under the UK Coronavirus Act (2020) giving police the authority to issue fines of up to £960 to those that did not comply.

After seven weeks of strict lockdown rules in the UK, outdoor recreation was amongst the first areas of daily life to experience a loosening of restrictions. In his televised speech to the British public on 10th May 2020, the British Prime Minister stated that, “We want to encourage people to take more and even unlimited amounts of outdoor exercise. You can sit in the sun in your local park, you can drive to other destinations, you can even play sports” (Johnson 2020).

It was not until the middle of June that restrictions began to be lifted more generally. Our analysis runs through to 15th June when many retail shops and public-facing businesses were allowed to re-open to the public.

### Google Mobility Reports Data

Evidence regarding the impact of the lockdown rules on the use of greenspace is provided by Google’s COVID-19 Community Mobility Reports (Google 2020). Using data from mobile devices running Google software enabled for location reporting, the Mobility Reports record changes in engagement in different activities over the lockdown period. The data is presented as a daily time series by region and records the percentage change in visits to numerous types of destination. Our focus is on the data provided on trips to parks which Google describe as including locations such as national parks, marinas, public beaches, dog parks, plazas and public gardens. Google also comment that the parks data does not include visits to “the general outdoors found in rural areas” (Google 2020).

This paper uses the Google time series for 86 regions in England spanning the period 15th February to 7th June 2020.^4^,^5^ Each data point in a time series indicates the park visitation observed on that day relative to activity levels observed in that region over a baseline period. The baseline period used by Google is the five weeks from 3rd January to 6th February 2020. In particular, a data point shows the percentage difference in visitations on that day relative to the median visitation observed for that same day of the week over the baseline period. Throughout this paper we refer to that measure as one of *park visitation change*.

**Fig. 1:**
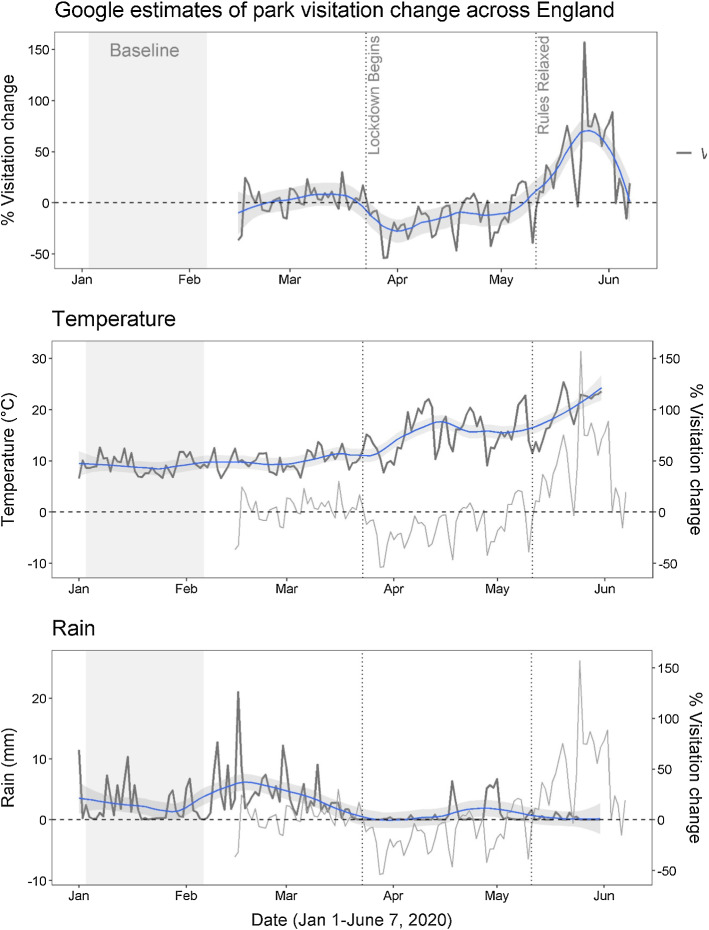
Google Mobility Reports time series for the UK (top panel) compared to temperature data (middle panel) and rainfall data (bottom panel)

The time series for England as a whole is shown in the top panel of Fig. 1, overlain with a smooth plot showing the central trend of the time series over the period. Observe that the visitation change data initially oscillates around an average value of 6.1%. In other words, the park visitation measured by Google over the period before the lockdown was around 6.1% higher than that measured over the baseline period. The impact of the enforcement of a strict lockdown on 23rd March appears to leave a clear signal. Over the seven weeks from 23rd March through to 10th May visitation change falls to around 17% of baseline levels. Likewise the relaxing of lockdown measures around 10th May, including the sanctioning of driving for engagement with the outdoors, coincides with a sharp upswing in parks visitation. On average over that last period of the time series visitation change is around 37% above baseline levels.

On first examination, the Google data appear to support the notion that outdoor recreation patterns in England were significantly affected by the lockdown rules. Google, however, caution against over-interpretation of the raw data (Google 2020). The baseline for the data (3rd January to 6th February 2020) was chosen as a period before widespread disruption from COVID-19. Even without the disruption of COVID-19 and the lockdown, we would expect outdoor recreation patterns to change from the winter months of the baseline to the spring and summer months of the lockdown.

The central and bottom panels of Fig. 1 over-plot the park visitation change time series with temperature and rainfall data for England.^6^ On both panels, a smooth of the weather data is provided to identify the central trend. Figure 1 reveals that the beginning of lockdown on March 23rd coincided with a well-defined change in the weather in England. After a very wet February and early March, the UK entered a prolonged dry spell. Temperatures also began to increase, starting in the low tens at the beginning of lockdown and climbing to the low twenties by the end of May. A reasonable expectation might be that outdoor recreation would increase with that warmer and dryer weather, an expectation that runs contrary to the sharp fall observed in the park visitation change time series at the beginning of the lockdown.

After an initial sharp fall, the visitation change data assumes a general rising trend that mirrors the rising temperature across England. It would be reasonable to assume that at least part of the differences in visitation seen over this period are attributable to the improving weather. In a similar vein, it is evident that visits respond to particular weather events. Down spikes in the Google data can be seen to coincide with significant rain events. Likewise some of the peaks in the visitation data appear to correlate with spells of warm weather.

In this paper, we take the patterns of change as suggesting that the story of greenspace use under lockdown in England can be broadly characterised as consisting of two distinct periods;

- *Strict Lockdown Rules (23rd March to 10th May)* Over the first period of lockdown the restrictions on the use of greenspace will have exerted downward pressure on recreation activity. We expect also that behavioural adjustments to avoid infection over this period will have further reduced demand relative to normal activity levels. The upward trend in visitation change after the initial sharp fall, may reflect improving weather conditions.
- *Relaxed Lockdown Rules (11th May to 15th June)* Entering this second period of lockdown, two things changed. First the rate of new cases had begun to fall, suggesting that England was past the peak of the virus and that the risk of infection was now falling, Perhaps more significantly restrictions on outdoor recreation were lifted. Both those factors will have acted to increase visitation to outdoor greenspace. That these increases in visitation are so substantial suggests that demand for greenspace may also have been inflated by the lack of alternative uses of leisure time coupled with a large segment of the population being freed from the time constraints of their normal working conditions^7^

## A Structural Model of Outdoor Recreation Activity: the ORVal Model

### A Brief Summary of the ORVal Model

The ORVal model is underpinned by the ORVal greenspace map, a detailed spatial dataset that describes the location and characteristics of accessible greenspace across England (Day and Smith 2016). The ORVal greenspace map identifies some 128,295 greenspace sites in England that could form the focus of a recreational trip. Each recreation site is described by its physical characteristics including its dimensions, landcovers, designations and points of interest.

Data to estimate the ORVal model was provided by the Monitor of Engagement with the Natural Environment (MENE) survey (Natural England 2017). Collected for the purposes of UK government National Statistics, the MENE survey provides a large, representative and random-location sample of adult (over 16 years of age) residents of England. The survey records trips to greenspace taken by each respondent over the seven days prior to the interview. For one randomly selected trip, the focus trip, the survey elicits detailed information including the location of the site visited and the mode of travel used to reach that destination.

The MENE survey runs throughout the year, sampling at least 800 respondents each week ensuring the data is temporally representative. ORVal was estimated from seven waves of data from 2009/10 through to 2015/16. In estimating ORVal, the destinations of focus trips in the MENE data were matched to the ORVal greenspace map and choice-based sampling used to draw 78,154 observations for the purposes of model estimation. Our econometric estimation corrects subsequently for the nature of the sample selection rule (Manski and Lerman 1977).^8^

Given the nature of the MENE data, the ORVal model progresses from the assumption that each day represents a recreation choice occasion on which individuals can select from a choice set comprising (1) not taking an outdoor trip, and then (2) an option for traveling to each site by car and (3) an option for each site visited on foot. As such, our econometric model takes the form of a repeated discrete-choice recreation demand model (Morey et al. 1993; Breffle and Morey 2000) where the repetition is over recreation decisions each day and the discrete choice is the decision over which of the options to select from the choice set.

One significant complication in estimating a recreation demand model for all recreation possibilities across an entire nation is the size of the choice set. In estimating the ORVal model we make use of techniques of importance sampling to select a choice set for each individual that provides us with reasonable power in identifying the parameters of the model (Guevara and Ben-Akiva 2013). Our subsequent estimating procedures make corrections for choice-set sampling (Daly et al. 2014).

Following standard practice the ORVal model is constructed from a linear specification of conditional indirect utility functions (McFadden 1973). For the option of not taking a trip to an outdoor recreation area (alternatively, to choose the outside good) utility is assumed to be a function of an individual’s characteristics (e.g., age, ethnicity, dog ownership, gender) the features of the particular day (e.g., the weather, time of the year, day of the week) and a set of spatial fixed effects defined by administrative regions at the level counties, unitary authorities and London Boroughs. More formally, the utility of the outside good, labelled option 0, for person $i$ on day $t$, is given by;

1 $$\begin{aligned} u_{i0t} & = v_{i0t} + \varepsilon_{i0t } \\ & = \varvec{x}_{it}\varvec{\beta}_{0} + \varepsilon_{i0t } \\ \end{aligned}$$

where $v_{i0t}$ is the modelled part of utility which is taken to be a linear function of the factors assumed to influence choice of the outside good, labelled $\varvec{x}_{it}$, and a set of parameters, $\varvec{\beta}_{0}$. Finally, $\varepsilon_{i0t }$ is an econometric error term.

A similar formulation is used to characterise options where recreation is chosen. These options are two-dimensional; they comprise both the choice of a greenspace destination and a mode of transport. In the ORVal model we assume that the utility from a site-mode combination is driven by two main factors; that site’s characteristics including its landcover (e.g., woodland, natural grass, saltmarsh), designations (e.g., national park, country park, nature reserve), points of interest (e.g., archaeological remains, historic buildings, playgrounds, car parking facilities) and, second the costs that the individual incurs in travelling to that site by a particular transport mode. In ORVal those calculations are expressed as a monetary travel cost, $tc_{ijq}$; that is to say the combined costs in time and money that individual $i$ incurs in traveling to site $j$ using mode $q$ (i.e. car or walk).^9^ Accordingly our model of site-mode utility is given by;

2 $$\begin{aligned} u_{ijqt} & = v_{ijqt} + \varepsilon_{ijqt } \\ & = \varvec{z}_{j}\varvec{\beta}_{1} - \gamma tc_{ijq} + \varepsilon_{ijqt } \\ \end{aligned}$$

where $v_{ijqt}$ is modelled utility for a site-mode option which is a linear function of a vector of site characteristics, labelled $\varvec{z}_{j}$, associated with a set of parameters, $\varvec{\beta}_{1}$. Utility is also determined by the travel costs of that site-mode option, $tc_{ijq}$ with associated parameter $\gamma$ interpretable as the marginal utility of income. Again, $\varepsilon_{ijqt}$ is an econometric error term.

Our estimating equations follow from the choice of distribution for the error terms, $\varepsilon_{i0t } \left( {\forall i,t} \right)$ and $\varepsilon_{ijqt} \left( {\forall i,j,q,t} \right)$. In the ORVal model we assume those errors are draws from a distribution in the generalised extreme value (GEV) family (McFadden 1978). More specifically, we assume that the errors are independent over individuals $\left( i \right)$ and time $\left( t \right)$ while allowing for the possibility of correlation in error terms across site-mode options belonging to the same, pre-defined similarity group. In ORVal, those similarity groups are identified by mode of transport (i.e. car, walk), the type of recreation site (i.e. park, path, beach) and the landcovers and land uses characterising a site (i.e. agriculture, allotment, church yard, moors and heath, natural grass, coastal, woods, wetlands, managed grass and fresh water). Site-mode options can be members of more than one group, with the degree of membership of an option in a landcover group being determined by the proportion of a site’s area under that landcover. A final, single-member group contains the outside option. Those particular assumptions lead us to the cross-nested logit model specification (Bierlaire 2006) in which the probability of a particular mode-site option is given by;

3 $$P_{ijqt} = \mathop \sum \limits_{n} \left( {\frac{{\left( {\mathop \sum \nolimits_{k} \mathop \sum \nolimits_{q} \alpha_{kqn} e^{{v_{ikqt} \lambda_{n} }} } \right)^{{1/\lambda_{n} }} }}{{\mathop \sum \nolimits_{l} \left( {\mathop \sum \nolimits_{k} \mathop \sum \nolimits_{q} \alpha_{kql} e^{{v_{ikqt} \lambda_{l} }} } \right)^{{1/\lambda_{l} }} }} \cdot \frac{{\alpha_{jqn} e^{{v_{ijqt} \lambda_{n} }} }}{{\mathop \sum \nolimits_{k} \mathop \sum \nolimits_{q} \alpha_{kqn} e^{{v_{ikqt} \lambda_{n} }} }}} \right)$$

Here $P_{ijqt}$ represents the probability that person $i$, chooses to visit site $j$ using mode $q$ in time period $t$. In Eq. (3) similarity groups are indexed by $n = 1, 2, \ldots , N$, $\alpha_{jqn}$ identifies the pre-determined membership of site-mode option $j, q$ to similarity group $n$ and $\lambda_{n} \left( {n = 1, 2, \ldots , N} \right)$ are parameters that capture the level of correlation in error terms for members of group $n$.

Equation (3) can be developed into a likelihood function for the observed choices and the model parameters, $\varvec{\beta}_{0}$, $\varvec{\beta}_{1}$, $\gamma$ and $\varvec{\lambda}$ estimated through methods of maximum likelihood. A full description of the development of the ORVal model, the parameter estimates and robustness testing is available in Day and Smith (2017).^10^

### Estimation of Recreation Activity and Welfare Using ORVal

Given it is based on a spatially and socioeconomically representative sample, ORVal can be used in exercises predicting recreation activity for the population of England. Estimating visits is relatively straightforward. Given an individual’s characteristics and their travel costs for each site-mode option, Eq. (3) can be used to predict the probability of them visiting some particular site using a particular transport mode on a particular day. In the analyses we present later, our focus is on predicting the number of visits to a region over a particular period of time. To estimate that for an individual using the ORVal model, one would simply sum the daily probabilities of visiting a site in that region where the probabilities would differ from day to day over that period on account of changing weather, day of the week and month of the year. To estimate total visits to the region over that period one would sum the result of that calculation for all adult residents of England.

The predictions reported in this paper make a number of simplifications to that calculation both to account for the availability of data and to manage the magnitude of the calculation task. First, our predictions are based on the populations of small-area statistical areas named Lower Super Output Areas (LSOAs) in England. The socioeconomic characteristics of LSOA residents was taken from the 2011 census and augmented with 2016 population estimates. We identify the population in each LSOA falling into 8 discrete groups defined by two key drivers of recreation engagement; socioeconomic segment and dog ownership.^11^ Taking averages of other sociodemographics, allows us to calculate daily visitation probabilities by group and LSOA.

To enable comparison with the observed Google mobility data, we require ORVal visitation predictions not only for the period of lockdown under both strict and relaxed rules but also for the period used as a baseline for the Google data; a total of 120 days. A second simplification we adopt in our analyses is to group days into categories and only estimate visitation probabilities for each category. In particular, we categorise days according to month and whether they fall on a weekday or a weekend. Our prediction period spans 6 months giving a total of 12 such day-month categories. In making visitation predictions we then use the Met Office daily weather data (see Sect. 3.2) to calculate the average weather experienced in each LSOA for every day-month category. Our most disaggregate visitation probabilities, therefore, constitute predictions for each day-month category from a socioeconomic group in an LSOA to a recreation site.

Aggregation to regional visit estimates on a particular day-month combination proceeds through a number of steps. First, for each socioeconomic group in an LSOA, we sum the visitation probabilities for that day-month combination across all sites in a region. Multiplying up by each group’s population in that LSOA and summing provides an estimate of visitation from that LSOA to the region. Repeating those calculations across each of the 32,844 LSOAs in England and summing the results provides ORVal’s estimate of visits to a region. Since we will have cause to refer to this calculation later, a more formal presentation is given by;

4 $$\hat{V}_{gmd} = \mathop \sum \limits_{r} \mathop \sum \limits_{s} N_{{s_{r} }} \mathop \sum \limits_{{j,q \in C_{g} }} P_{{s_{r} jqmd}}$$

where $\hat{V}_{gmd}$ is the ORVal estimate of visits to region $g$ on the particular day-month combination given by the index $md$ where $m$ indexes months and $d \in \left\{ {weekday, weekend } \right\}$; $r$ indexes LSOAs while $s$ indexes the set of socioeconomic groups, such that $N_{{s_{r} }}$ is the number of individuals in group $s$ living in LSOA $r$; $C_{g}$ is the set of site-mode options in region $g$ and $P_{{s_{r} jqmd}}$ is the ORVal estimate of the group-day-month probability of visiting site $j$ by transport mode $q$.

One useful property of GEV models is that there exists a simple closed-form expression for the expectation of the maximum utility a respondent might expect to derive from being able to choose an option from their choice set. In the case of the cross-nested logit model that expression amounts to;

5 $$W_{it} \left( {C } \right) = {\text{ln}}\left( {e^{{v_{i0t} }} + \mathop \sum \limits_{n} \left( {\mathop \sum \limits_{j,q \in C} \alpha_{jqn} e^{{v_{ijq} \lambda_{n} }} } \right)^{{1/\lambda_{n} }} } \right) + \mu$$

where $W_{it} \left( {C } \right)$ is the expectation of maximum utility realised by individual $i$ in time period $t$ given the opportunity to choose from the set of site-mode options in the choice set $C$, and $\mu$ is the Euler-Mascheroni constant (that takes a value of approximately 0.5772).

It follows that the expected level of welfare change that an individual would experience if the nature of their choice set were to change can be estimated from (Small and Rosen 1981);

6 $$\Delta W = \frac{1}{\gamma }\left( {W_{it} \left( {C^{\prime}} \right) - W_{it} \left( C \right)} \right)$$

where $C$ is the original choice set and $C^{\prime}$ is the changed choice set. In simple terms, Eq. (6) describes the analyst’s best estimate of how an individuals’ utility will change as a result of changes in the choice set with that quantity translated into money terms by dividing through by the marginal utility of income, $\gamma$.

In this paper, the choice set restriction explored is the one created by the strict lockdown rules where individuals were prohibited from travelling to outdoor recreation sites by car. As with our visit calculations, arriving at welfare estimates for such changes for the whole of England requires aggregating up from group-day-month welfare estimates calculated at the LSOA scale.

## Calibrating ORVal to the Google Mobility Data

### Uncalibrated ORVal Predictions of Recreation Activity Under the Lockdown Rules

**Fig. 2:**
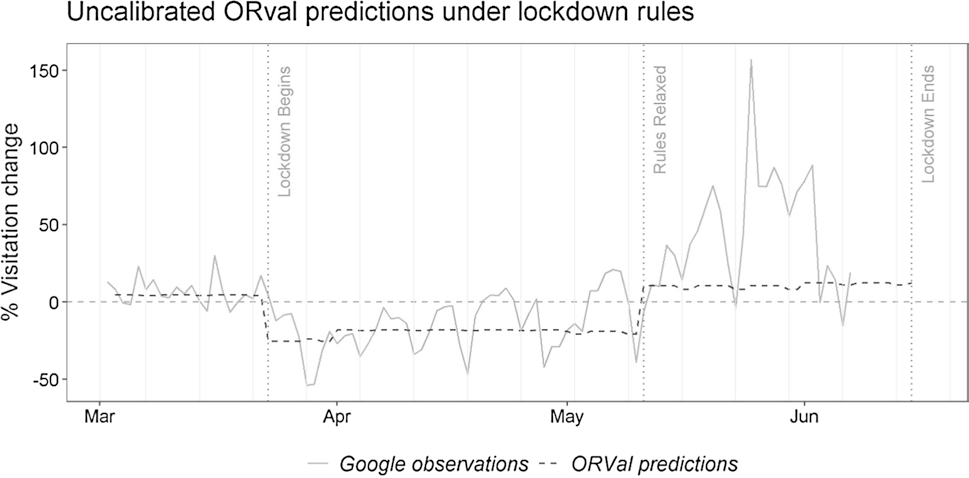
ORVal prediction of park visitation change in England under lockdown rules compared to Google time series for England

Using Eq. 4, we generate daily predictions of recreation activity over the lockdown, simulating the lockdown rules by removing the option of driving to greenspace from each individual’s choice set over the period of strict lockdown rules and returning those options to the choice set over the period of relaxed lockdown rules. In order to draw comparison with the Google mobility data, these ORVal predictions must be expressed in terms of visitation levels relative to the baseline period (3rd January to 6th February 2020). Accordingly, we also estimate visitation to each region during the baseline period, quantites we denote $\hat{V}_{{gd^{0} }}$.^12^ Daily ORVal predictions of relative regional visitation, compatible to those in the Google data can then be calculated according to $\hat{V}_{gmd} /\hat{V}_{{gd^{0} }}$. Figure 2 plots out these ORVal prediction of visitation change over the lockdown period comparing them to those in the Google Mobility data.

In interpreting Fig. 2, it is worth noting some caveats regarding the validity of a straight comparison of the two data series. First, there is not perfect congruence in the set of locations considered as outdoor recreation destinations. Google’s estimates, for example, ignore recreational use of countryside paths, trips that are included in ORVal estimates. Second Google’s data reports on visitors to regions irrespective of their home location while ORVal is restricted to visits from residents of England. Third, ORVal predicts day trips to greenspace locations but the Google data does not distinguish between day trips and trips made while staying overnight away from home. Fourth, the Google data records visits by individuals carrying mobile devices enabled for location reporting, a group which does not necessarily represent the adult population of England whose behaviour is modelled by ORVal. As a final comment, we note the fact that the Google data is reported in relative terms. Accordingly, our comparisons are predicated on the assumption that changes in recreation behaviour in the areas of incongruence between the two data series experience the same relative changes as those where they overlap.

Observe that a sharp step down in the ORVal predictions is evident as the strict lockdown rules are brought into force and the option of driving is removed from choice sets. The predicted time series steps up again when the recreation activity rules are relaxed and continues on to the date at which the general lockdown began to be lifted on 15th June. Unfortunately, at the time of writing Google had not released its mobility data for the period between 7th June and that date.

The parameters of the ORVal model are estimated from the observed recreation behaviour of the English population under normal conditions. The fact that over the strict lockdown period the ORVal predictions are relatively lower than the baseline arises, therefore, purely on account of the removal of the option to travel by car. The predictions do not make adjustment for the other possible drivers of visitation change under lockdown. All the same, the ORVal time series does a reasonable job at defining the central trend of the Google data over this period.

In the period of relaxed lockdown rules, the ORVal predictions rise to a level of around 10% above the baseline. Again these predictions simply reflect normal recreation in May and June which tends to exceed that in the winter months of the baseline. Notice, however, that over this second period of lockdown the ORVal predictions lie well below the central trend of the Google data. Clearly, the recreation behaviour recorded in the Google mobility data over this period cannot be explained solely on account of normal variation in recreation activity across the year.

A further clear pattern of difference between the Google time series and the ORVal time series concerns recreation activity over weekends. In Fig. 2 the Saturday of each weekend is marked by a light grey vertical line. Recall that both time series are expressed in measures of visitation relative to the baseline. Accordingly, while ORVal predicts weekend rates of visitation to be substantially higher than midweek visitation, it does so both in the baseline period and in the periods of lockdown. Indeed, for ORVal, the ratio of the weekday and weekend predictions to their counterparts in the baseline remain relatively constant for both lockdown periods. The same is not true of the Google time series. Following the commencement of lockdown, that data series is characterised by a regular pattern of down spikes coinciding with weekend periods. Since those same down spikes are not evident in early March, they are suggestive of a systematic change in behaviour during the lockdown. In particular, lockdown appears to have resulted in a relative redistribution of visits across the week with comparatively more trips being taken on weekdays when compared to weekends. Such changes are compatible with a relaxing of leisure time constraints amongst workers normally limited to weekend periods for their outdoor recreation.

### Calibrating ORVal Predictions

Figure 2 makes clear that the use of greenspace over the lockdown was not simply normal patterns of recreation behaviour constrained by the lockdown rules. Indeed, differences between the ORVal and the Google time series provide insights into the scale of the demand shifts precipitated by the various other factors impacting on greenspace use over this period. Accordingly, the next step in our analysis is to use those observed differences to estimate parameters for the ORVal model that capture the demand shifts experienced under lockdown.

Within the ORVal model, a demand shift parameter, $\tilde{\beta }$, can be specified as a fixed factor entering the utility function for the outside good. Adding that parameter to Eq. 1 we get;

7 $$v_{i0t} = \varvec{x}_{it}\varvec{\beta}_{0} + \tilde{\beta }$$

If $\tilde{\beta }$ takes a negative (positive) value then the utility of the outside good falls (increases) and visiting greenspace is relatively more (less) attractive.

Of course our comparison of the Google and ORVal time series suggests that the level of demand shift differs from the first period of lockdown to the second and, during each of those periods, from weekdays to weekends. Accordingly, we seek to estimate four demand shift parameters, $\tilde{\beta }_{Td}$ where $d \in \left\{ {weekday,weekend} \right\}$ and $T$ indexes periods of the lockdown; that is, $T \in \left\{ {T^{1} ,T^{2} } \right\}$.

We build our estimating equations from the basic assumption that, augmented by the true shift parameters, the ORVal model provides unbiased estimates of the daily visits to a region’s greenspaces. Recall from Eq. 4 that to reduce computational burden, predictions of visitation on day $t$ are approximated by an estimate specific to the month of $t$ $\left( {m_{t} } \right)$ and whether $t$ is midweek or on a weekend $\left( {d_{t} } \right)$. The calibrated ORVal estimate of visitation to region $g$ on day $t$, therefore, can be denoted $\hat{V}_{{gm_{t} d_{t} }} \left( {\tilde{\beta }_{{T_{t} d_{t} }} } \right)$ where $T_{t}$ indicates the period of lockdown in which day $t$ falls. The actual number of visits, $V_{gt}$, differs from the ORVal estimate on account of myriad factors that we relegate to a mean-zero error term. According to this model, the Google and ORVal estimates of relative visitation to region $g$ on day $t$ of lockdown period $T$ are related according to the equation;

8 $$Y_{gt} = \frac{{V_{gt}}}{{V_{{gt^{0}}}}} = \frac{{\hat{V}_{{gm_{t} d_{t}}} \left({\tilde{\beta}_{{T_{t} d_{t}}}} \right) + \epsilon_{{gmd_{t}}}}}{{\hat{V}_{{gd_{t}^{0}}} + \epsilon_{{gd_{t}^{0}}}}}$$

where $Y_{gt}$ is the visitation change observed by Google, $\bar{V}_{{gt^{0} }}$ is the median level of visitation to region $g$ on the same day of the week as $t$ during the baseline and $\hat{V}_{{gd_{t}^{0} }}$ is ORVal’s prediction of visits during the baseline on a day equivalent to that identified by $d_{t}$.

We progress by assuming that the error terms in Eq. (8) are independent draws^13^ from a mean-zero normal distribution with variance $\sigma^{2}$. It follows that the right-hand-side of Eq. 8 amounts to a ratio of normal variates with identical variance but different means. Such a ratio is a Cauchy distributed variate with probability density function $p_{Y} \left( {y; \mu_{1} ,\mu_{2} ,\sigma^{2} } \right)$, where $\mu_{1}$ is the mean of the normal variate in the numerator and $\mu_{2}$ the mean of the normal variate in the denominator (see Hinkley 1969 for the exact functional form of this probability). Given values for the demand shift parameters and the variance parameter, $\sigma^{2}$, therefore, we can calculate the probability of observing each data point in the Google time series according to $Prob\left[ {Y_{gt} |\tilde{\beta }_{{T_{t} d_{t} }} ,\sigma^{2} } \right] = p_{Y} \left( {Y_{gt} ;\hat{V}_{{gm_{t} d_{t} }} \left( {\tilde{\beta }_{{T_{t} d_{t} }} } \right),\hat{V}_{{gd_{t}^{0} }} ,\sigma^{2} } \right)$. The demand shift parameters can then be estimated by solving the maximum likelihood problem;

9 $$\mathop {\hbox{max} }\limits_{{\tilde{\beta }, \sigma^{2} }} {\text{ln}}L\left( {\varvec{Y}|\tilde{\varvec{\beta }},\sigma^{2} } \right) = \mathop \sum \limits_{g} \mathop \sum \limits_{t} \ln p_{Y} \left( {Y_{gt} ; \hat{V}_{{gd_{t}^{0} }} ,\hat{V}_{{gm_{t} d_{t} }} \left( {\tilde{\beta }_{{T_{t} d_{t} }} } \right),\sigma^{2} } \right)$$

where $\varvec{Y}$ is the vector of Google parks visitation observations for each region over each day of the lockdown period and $\tilde{\varvec{\beta }}$ is the vector of demand shift parameters to be estimated.

The possibility exists that behavioural responses to lockdown may have differed across England. To explore that possibility we expand Eq. 9 into a latent class regression analysis (Wedel and DeSarbo 1994). In this analysis we assume that the English population consists of a finite set of unobserved sub-populations or classes, indexed by $h = 1, \ldots , H$ with each class characterised by different demand-shift parameters, $\tilde{\varvec{\beta }}^{h}$. The unobserved size of the population in each class is given by a group membership proportion $\pi_{h}$ (with $\sum\nolimits_{h} {\pi_{h} } = 1$).

The log likelihood for the latent class regression is given by;

10 $$\ln L\left( {\tilde{\varvec{\beta }}^{1} , \ldots ,\tilde{\varvec{\beta }}^{\varvec{H}} ,\varvec{\tau},\varvec{\sigma}^{2} } \right) = \mathop \sum \limits_{g} \ln \mathop \sum \limits_{h} \pi \left( {\tau_{h} } \right)\mathop \prod \limits_{t} p_{Y} \left( {Y_{gt} ; \hat{V}_{{gd_{t}^{0} }} ,\hat{V}_{{gm_{t} d_{t} }} \left( {\tilde{\beta }_{{T_{t} d_{t} }}^{h} } \right),\sigma_{h}^{2} } \right)$$

where class membership probability, $\pi \left( {\tau_{h} } \right)$, is specified as a function of a parameter $\tau_{h}$ according to ${ \exp }\left( {\tau_{h} } \right)/\mathop \sum \limits_{k} { \exp }\left( {\tau_{k} } \right)$. The parameters to be estimated include the demand shift parameters for each class, $\tilde{\varvec{\beta }}^{\varvec{h}}$, the class membership parameters $\varvec{\tau}= \left[ {\tau_{1} , \ldots ,\tau_{H} } \right]$ and the class variance parameters $\varvec{\sigma}^{2} = \left[ {\sigma_{1}^{2} , \ldots ,\sigma_{H}^{2} } \right]$.

Following standard practice (Nylund-Gibson and Choi 2018), the log likelihood in Eq. 10 was maximised over a series of different assumptions regarding the number of classes, with a four-class model being chosen as the model delivering the best fit according to the Bayes Information Criterion (BIC). Parameter estimates from that model are reported in Table 1.

**Table 1:** Parameter estimates from a four-class latent class regression model identifying parameters for the ORVal recreation demand model identifying patterns of demand-shift observed under lockdown

Parameter	Latent class			
	Class 1	Class 2	Class 3	Class 4
Class membership				
$\tau_{h}$	0	0.559 (0.375)	− 0.086 (0.403)	0.111 (0.386)
Probability $\left( {\pi_{h} } \right)$	0.209	0.365	0.192	0.234
Demand-shift params $\left( {\tilde{\beta }_{Td}^{h} } \right)$				
Strict rules				
Weekday	− 0.310*** (0.028)	− 0.154*** (0.017)	− 0.127*** (0.031)	0.060** (0.030)
Weekend	− 0.001 (0.023)	0.204*** (0.020)	0.082*** (0.025)	0.474*** (0.025)
Relaxed rules				
Weekday	− 0.646*** (0.027)	− 0.369*** (0.015)	− 0.325*** (0.028)	− 0.265*** (0.026)
Weekend	− 0.559*** (0.024)	− 0.141*** (0.012)	− 0.154*** (0.021)	− 0.048*** (0.022)
$\sigma_{k}$	0.511*** (0.054)	0.285*** (0.024)	1.402*** (0.142)	0.356*** (0.042)

Table reports the coefficient estimate with the standard error below in brackets. Coefficients significant at the 90% level are highlighted with *, those at the 95% level with ** and those at 99% at ***

**Fig. 3:**
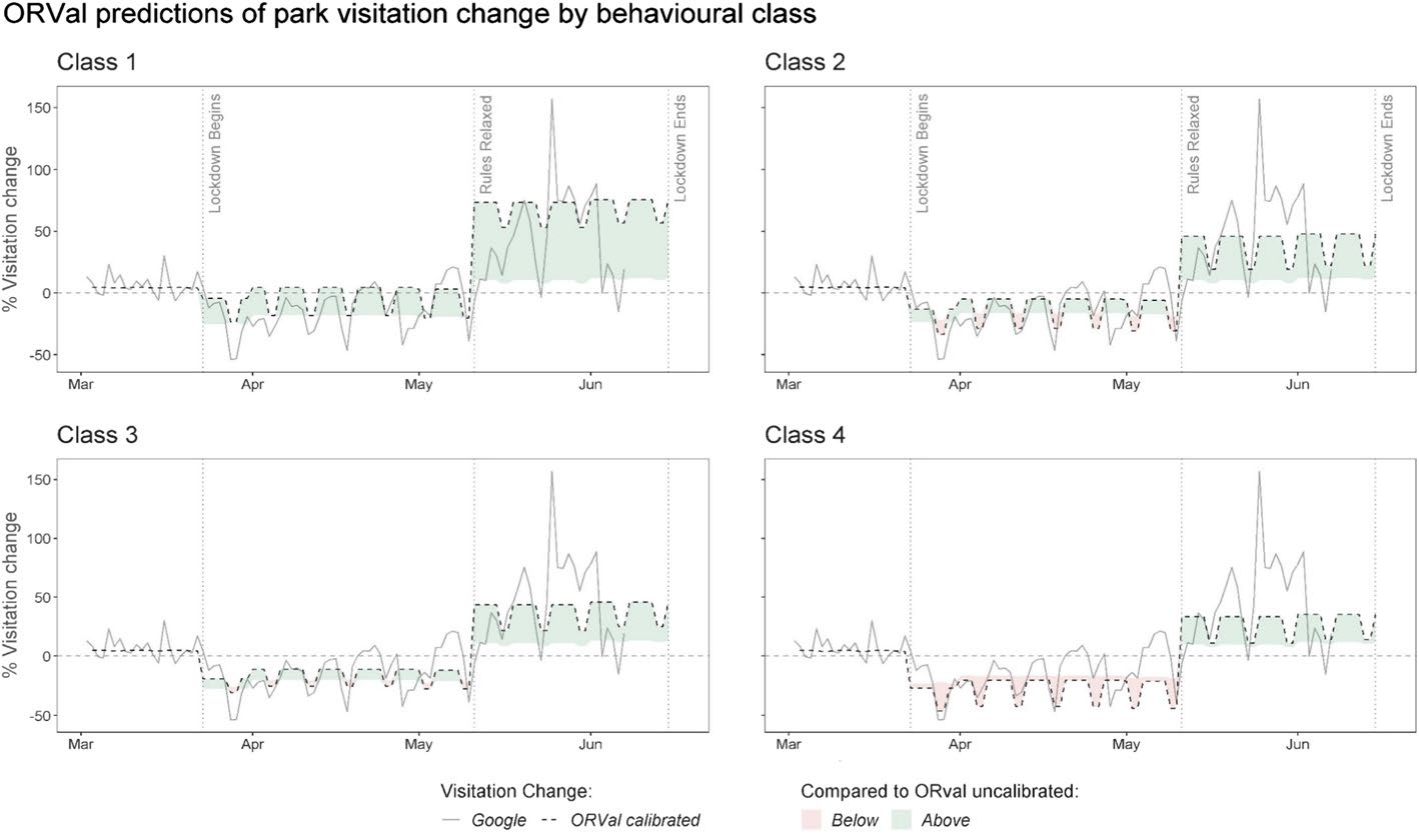
Calibrated ORVal predictions of park visitation change for each behavioural class

The a priori class membership probabilities, $\pi_{h}$, suggest a fairly even distribution of membership over the four classes ranging from 19.2% in Class 3 up to 36.5% in Class 2. To help in the interpretation of the demand-shift parameters, Fig. 3 plots out the implied park visitation change time series associated with each different class. In that Figure, comparison is made to the uncalibrated ORVal predictions; a time series which assumes that the only change experienced during the lockdown was the imposition of restrictions on recreation activity. The shaded areas show how demand for trips to the outdoors for each class differs from that reference level. Areas shaded in green show periods where demand for trips to the outdoors exceeded the reference, those in red where demand fell below the reference.

The first thing to note from Fig. 3 is that for each class the demand-shift parameters distinguish a change in relative preferences for recreation on weekdays as compared to weekends. Compared to the reference (ORVal’s uncalibrated time series), over the lockdown relatively more trips are taken during the week and relatively less on weekends; possibly a result of an easing of leisure-time constraints on furloughed workers. Also observe from Fig. 3 that when the lockdown rules were relaxed, levels of demand for all four classes substantially exceed reference levels. That pattern possibly reflects a substitution effect as people turned to outdoor recreation in lieu of access to other prohibited leisure activities. It might also reflect an increasing propensity to engage in outdoor activities as the risks of infection diminished.

Considering the Class 1 predictions, notice that over both periods of lockdown the time series exceeds that of the uncalibrated reference; the net effect of the demand shifters for this class is to increase use of the outdoors. Indeed, Class 1 represents the sub-population whose demand for the outdoors increased most substantially under lockdown.

The patterns of recreation activity expressed by populations in Class 2 and Class 3 are reasonably similar. In both, over the period of strict lockdown rules, recreation activity tracks reference behaviour, differing primarily in the redistribution of visits from weekends to weekdays. That redistribution effect is somewhat more substantial for Class 2 populations. Over this first period of lockdown, it appears that for Classes 2 and 3 the demand-reducing effect of virus-exposure risk and the demand-increasing effect of restrictions on alternative leisure options are either small or act to cancel each other out. After the relaxation of lockdown rules, both classes exhibit a similar and substantial upward shift in demand for recreation, though the redistribution of trips from weekends to weekdays remains more pronounced in Class 2.

Class 4 are the only population to exhibit levels of recreation activity than are consistently lower than the reference. For these populations the period of strict lockdown saw engagement with the outdoors fall below that which might be expected just from the restrictions on driving to recreation locations. After the relaxation of that rule, Class 4 populations expanded their demand for outdoor recreation above reference behaviour, but considerably less so than the other populations.

### Spatial Distribution of Behavioural Classes

While the group membership probabilities of Table 1 provide an indication of the mix of different behavioural classes across England, it is also possible to derive an estimate of the specific mix characterising visits to each region of the Google data. Using Bayes theorem, the posterior probability that the observed visitation data for region $g$ results from populations expressing the Class $h$ recreation pattern of recreation activity is;

11 $$\pi_{h}^{g} = Prob\left[ {Y_{g}\,from\,h} \right] = \frac{{\pi_{h} \mathop \prod \nolimits_{t} p_{Y} \left( {Y_{gt} |\tilde{\beta }_{{T_{t} d_{t} }}^{h} } \right)}}{{\mathop \sum \nolimits_{k} \pi_{k} \mathop \prod \nolimits_{t} p_{Y} \left( {Y_{gt} |\tilde{\beta }_{{T_{t} d_{t} }}^{k} } \right)}}$$

Calculating such posterior probabilities for each class, we arrive at a set of estimates $\left( {\pi_{h}^{g} ;\, h = 1, \ldots , H} \right)$ that we interpret as representing the proportions of visitors from each class contributing to recreation activity in region *g*. Accordingly, we refer to those quantities as the class shares for a region’s visits. Our objective is to use these class shares to determine the class most likely to represent the recreation behaviour of the population of each LSOA. Knowing those classes allows us to calibrate the ORVal model by assigning the appropriate demand-shift parameters to the choice equations for residents of each LSOA. ORVal can then be used to derive estimates of recreation activity and welfare changes under lockdown condiations.

One approach to assigning classes to LSOAs would be to identify the region in which an LSOA is located and ascribe it the class for that region with the highest visit share. The intuition here is that the majority of visits from an LSOA, $r$, will be to the region in which it is located, $g_{r}$, such that our best guess of the behaviour class of an LSOA’s population will be that most frequently observed in visits to $g_{r}$. Of course, that calculation ignores the fact that residents of an LSOA may also visit other regions, such that information about the behaviour class of an LSOA is also contained in the class shares of visits to those other regions. To make use of that information, we make an initial guess at the trips taken by residents of LSOA, $r$, to each region, $g$,^14^ and use those to calculate the proportion of visits from $r$ that choose $g$ as a destination. Using these proportions as weights, we calculate the weighted sum of the class shares for each region’s visits, to arrive at our best guess of the class shares characterising $r$. We assign $r$ the class exhibiting the highest class share.

**Fig. 4:**
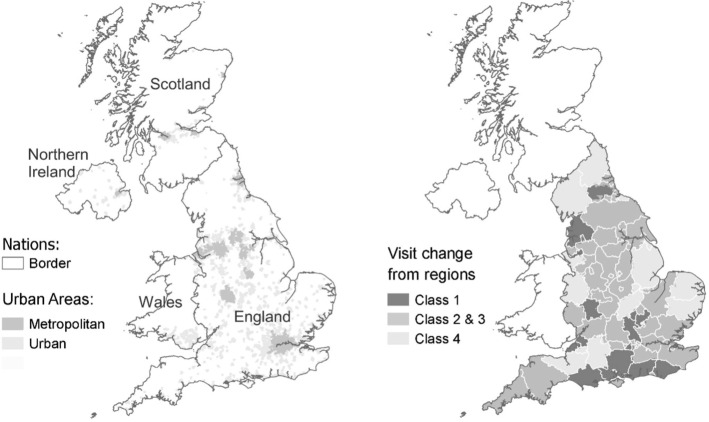
Distribution of different patterns of park visitation change from regions of England by four behavioural classes

Figure 4 maps out the classification of LSOAs in England to different classes. To simplify presentation and reflect their similarity, areas in Class 2 and 3 are presented in the same shade. While the data is plotted at the LSOA scale, as might be expected, the pattern of class membership broadly follows the regions upon which the data analysis is based. Those regions are outlined in white and close inspection reveals that our classification procedure allots some LSOAs along region borders to a different behaviour class to LSOAs in the region interior.

There exists some interpretable spatial pattern in the distribution of class membership described in Fig. 4. For instance, all the major metropolitan areas of England exhibit Class 2 and 3 behaviour changes (expected activity under strict lockdown, much increased activity under relaxed lockdown). In addition, Class 1 behaviour changes (increased activity under strict lockdown, greatly increased activity under relaxed lockdown), show clear patterns of regional clustering most notably along the south coast and central-south region of England. We suspect that these patterns reflect regional differences in the perceived and actual risks of exposure to the virus. Areas exhibiting Class 4 behaviour changes (reduced activity under strict lockdown, increased activity under relaxed lockdown) are largely located in relatively remote and rural areas of England. That pattern would be commensurate with locations whose workforces are primarily engaged in the food production sector; an occupation classed as essential in the lockdown and not subject to restriction under the lockdown rules.

## Predicting Recreation Activity and Welfare Under Lockdown

### Actual Lockdown Rules

**Fig. 5:**
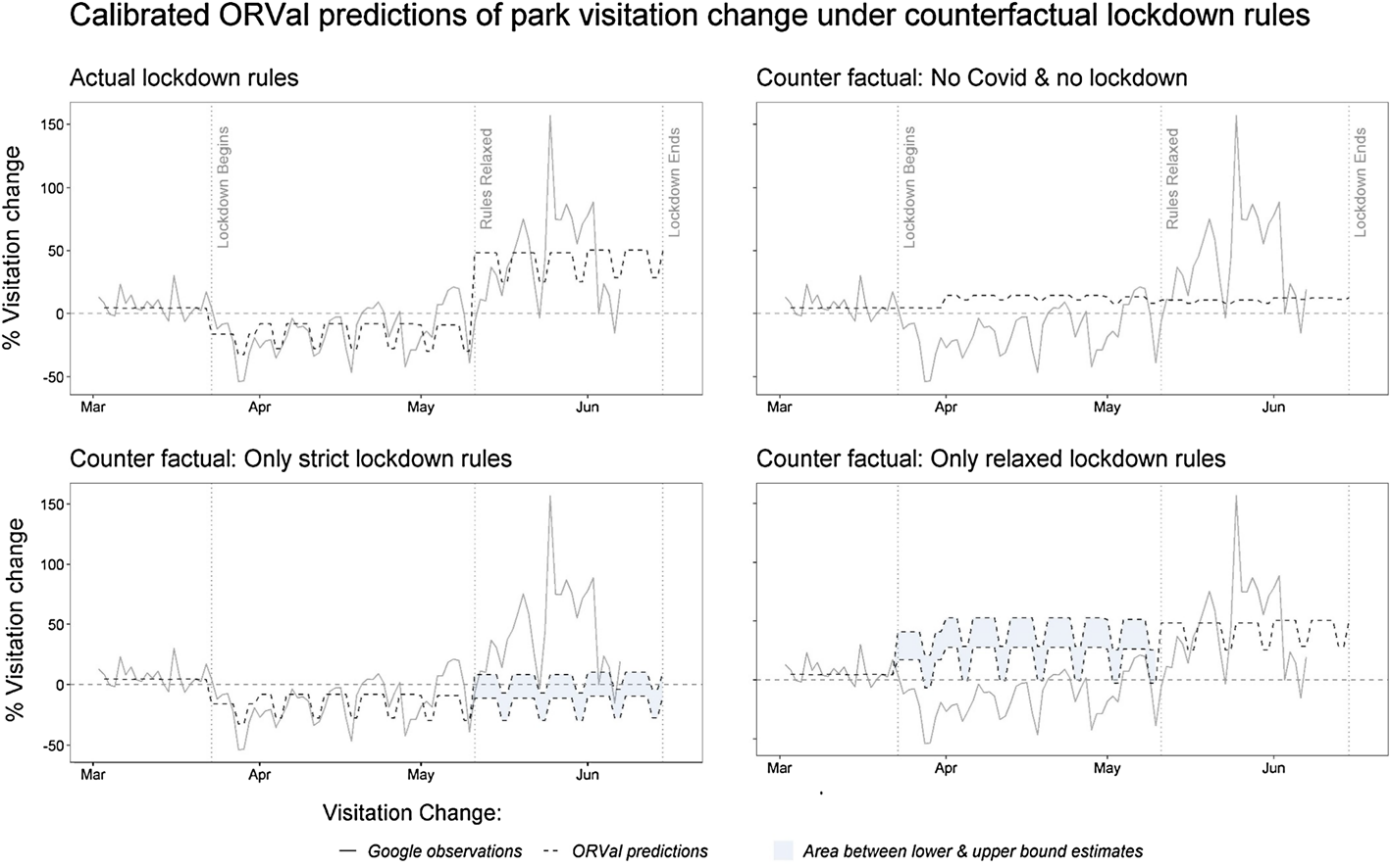
Calibrated ORVal predictions of park visitation change under actual and counterfactual lockdown rules

The top left panel of Fig. 5 presents ORVal’s predictions of visitation change for England once the recreational choices of residents of each LSOA have been adjusted with the demand shifters for their estimated behaviour class. Applying the methods described in Sect. 4.2, we can now use this calibrated version of the ORVal model to estimate levels of recreation activity under lockdown and the economic welfare generated from that activity. We take as our point of comparison the recreation activity that would have occurred absent the COVID-19 pandemic and the resulting lockdown. Those predictions are made by running out the uncalibrated and unrestricted ORVal model, a times series that is presented in the top right hand panel of Fig. 5. A summary of value and visit estimates under the lockdown and under the counterfactual of normal conditions are presented in Table 2.

**Table 2:** Comparison of ORVal estimates of total recreation value and visits for England under the COVID-19 lockdown (23 March to 15 June) compared to those under normal conditions

Scenario	Visits (millions)			Value of greenspace (£mill 2016)
	Car	Walk	All	
Normal conditions				
Strict period	168.6	198.8	367.4	£3238
Relaxed period	119.9	141.4	261.4	£2303
Total	288.5	340.2	628.7	£5541
Actual lockdown rules				
Strict period	–	275.0	275.0	£2338
Relaxed period	152.9	179.9	332.8	£3053
Total	152.9	454.9	607.8	£5390
Difference in total				
Absolute	− 135.6	114.8	− 20.9	− £150.89
Relative	− 47.0%	33.7%	− 3.3%	− 2.7%

The estimates in Table 2 are for visits and values aggregated over all English residents over (1) the seven weeks of strict lockdown rules, (2) the five weeks of relaxed lockdown rules (extended beyond the Google time series to the general loosening of lockdown restrictions on 15th June) and (3) totalled over both periods. The top part of the table reports those quantities under normal conditions, the middle part under the COVID-19 lockdown and the bottom part differences between the two.

Consider first the changes in estimated visits. Where normally we would expect some 168.8 million trips to the outdoors taken by car, such trips were prohibited over the 7 weeks of strict lockdown rules. What the ORVal estimates reveal is that individuals responded to those restrictions through substituting to trips taken on foot. In the period of strict lockdown, the calibrated model estimates that 275.0 million trips were taken to greenspaces on foot, an almost 40% increase over the 198.8 million expected under normal conditions.

**Fig. 6:**
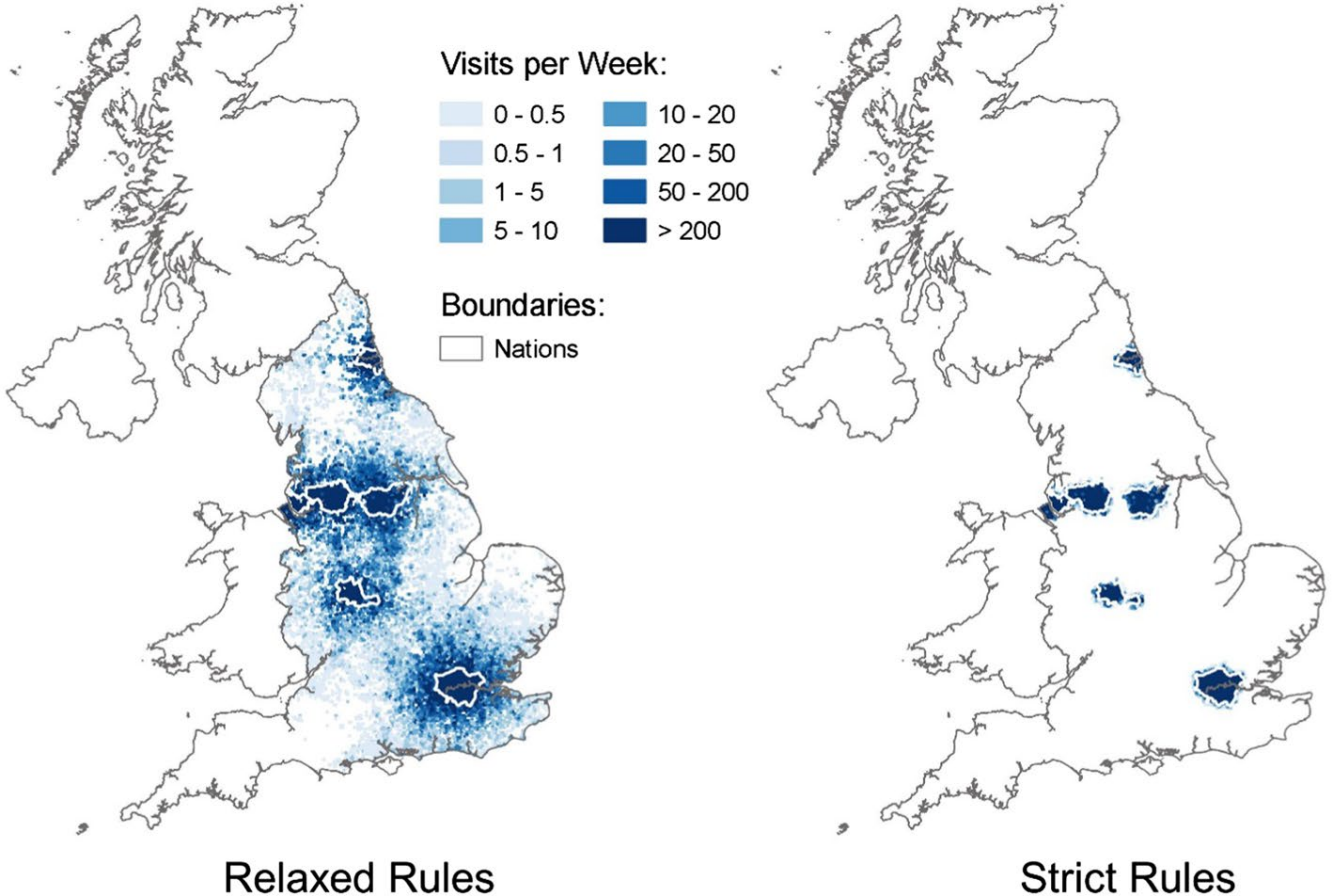
Weekly visits from England’s major metropolitan areas under normal conditions and under strict lockdown rules

Figure 6 illustrates how recreation behaviour changed across England in this period. The left-hand panel plots out ORVal estimates of the spatial distribution of weekly visits taken by residents of the major metropolitan areas of England under normal conditions. The right hand panel contrasts that with the distribution of visits under the strict lockdown rules. Prohibited from driving, outdoor recreation activity refocused on local greenspaces.

Once the restrictions on driving were lifted, the effects of the demand-shifts evident in the Google data become clear. Visits by both car and on foot increase, resulting in levels of recreation visits that are some 27.5% above those expected under normal conditions.

The story of outdoor recreation under lockdown is one in which people offset the restrictions on driving to recreation sites by switching to walking to greenspaces local to their homes. That behaviour along with an upward shift in demand for recreation resulted in the overall number of visits to the outdoors over the lockdown period being little changed from that under normal restrictions. Our calculations of welfare change suggest that the cost of lockdown on welfare derived from greenspaces was negligible, dropping by £150 million or some 2.7% of that realised under normal conditions.

### Counterfactual Lockdown Rules

A second set of analyses that are possible with the ORVal model use the calibration parameters to explore the visit changes and welfare consequences that might have arisen should alternative rules on recreation have been instituted in the lockdown. Here we consider two such counterfactuals. The first is a counterfactual where the strict lockdown rules prohibiting driving to greenspaces were extended over the whole period from March 23rd to June 15th. The second is a counterfactual in which no restrictions were imposed on recreation activity over lockdown.

Time series describing recreation activity under those two counterfactuals are presented in the bottom panels of Fig. 5. Observe that in the strict lockdown counterfactual we are projecting behaviour out over the second period of the lockdown under rules for which we do not have observations from the Google mobility data against which to calibrate. Two assumptions are possible. First that the demand shift parameters characterising behaviour under the strict lockdown period continue to characterise behaviour under the extension of those rules into the second period. Alternatively, that the second period might be characterised by the demand shift parameters charactering recreation behaviour during that second period under the relaxed rules. Since the demand shift parameters of the second period are universally more positive than those for the first period, those two assumptions suggest lower and upper bound estimates of possible behaviour under the strict lockdown counterfactual. Those bounds are traced out in the plot of Fig. 5 with the grey shaded area demarking the paths lying between those bounds. Similar arguments lead to bounds on the recreation activity over the first period in the relaxed lockdown rules counterfactual. These too are shown in Fig. 5.

Summary details of recreation visits and values under the strict lockdown counterfactual are presented in Table 3.

**Table 3:** Strict Lockdown Counterfactual: Comparison of ORVal estimates of total recreation value and visits for England under the COVID-19 lockdown compared to a counterfactual in which strict lockdown rules were imposed for the whole time period

Scenario	Visits (millions)			Value of greenspace (£mill 2016)
	Car	Walk	All	
Actual lockdown rules	152.9	454.9	607.8	£5390
Only strict lockdown (lower and upper bound)	0	496.1 (472–520)	496.1 (472–520)	£4249 (4016–4483)
Difference: Absolute	− 152.9	41.2 (17.5–64.9)	− 111.7 (−135 to − 88)	− £1141 (− 1374 to − 907)
Relative	− 100.0%	9.1% (3.8 –14.3)	− 18.4% (− 22 to − 14)	− 21.2% (− 25.5 to − 16.8)

In that table, we present estimates that are averages of those for the lower and upper bounds and contrast those with estimates of visits under the lockdown under the actual lockdown rules.

Not surprisingly, maintaining the rule prohibiting driving to outdoor recreation locations has the effect of suppressing engagement with greenspaces. The ORVal model predicts some expansion of walking in the second period of lockdown to compensate for the continuing restrictions on driving opportunities. All the same, maintaining strict rules on recreation over the whole lockdown results in an estimated 18.4% reduction in visits to the outdoors compared to those estimated under the actual lockdown rules. In terms of welfare, the stricter rules impose a welfare cost on English residents; the value flow realised from greenspace access falls by some £1.14 billion. Viewed the other way, the government’s decision to relax the rules on outdoor recreation activity delivered a £1.14 billion welfare boost to residents of England.

Table 4 provides an identical analysis for outcomes under the relaxed rules counterfactual in which the lockdown proceeded without restrictions on outdoor recreation activity.Under the relaxed-rules counterfactual ORVal predicts an expansion of recreation activity. Visits to the outdoors are some 24.2% greater than those estimated under the actual lockdown rules. Again that translates into changes in the economic value of greenspace. A lockdown with no restrictions on recreation activity increases the estimates of the welfare benefits of greenspace access by some £1.47 billion. Viewed the other way, English residents suffered a welfare cost of £1.47 billion as a consequence of the government’s decision to restrict recreation activity over the first period of the COVID-19 lockdown.

**Table 4:** Relaxed Lockdown Counterfactual: Comparison of ORVal estimates of total recreation value and visits for England under the COVID-19 lockdown compared to a counterfactual in which recreation activity was unrestricted for the whole time period

Scenario	Visits (millions)			Value of greenspace (£mill 2016)
	Car	Walk	All	
Actual lockdown rules	152.9	454.9	607.8	£5390
Only relaxed lockdown (lower and upper bound)	346.8 (328–366)	408.0 (386–430)	754.7 (714–796)	£6857 (6419–7296)
Difference: Absolute	193.9 (175–213)	− 47.0 (− 69.3 to − 24.7)	146.9 (106–188)	£1467 (1029–1906)
Relative	126.8% (115–139)	− 10.3% (− 15.2 to − 5.4)	24.2% (17–31)	27.2% (19.1–35.4)

## Concluding Remarks

Using analytical methods that leverage Google Mobility data and the predictive powers of the ORVal model, this paper explores how the COVID-19 lockdown in England changed how people engaged with greenspace and impacted on the economic value they derived from those interactions. We find strong evidence to support the contention that greenspace became a significant source of welfare for citizens at a time when opportunities for alternative uses of leisure time were even more seriously curtailed. One key change identified by our analysis is that the lockdown rules forced citizens to get out of their cars and walk. Trips to greenspaces by car fell by 47% over the whole lockdown period with an attendant 34% rise in trips taken on foot. Increased engagement in outdoor recreation (particularly in the second period of lockdown) coupled with this substitution behaviour meant that, despite the restrictions citizens maintained value flows from greenspace over the lockdown comparable to those they would have enjoyed over that same period under normal conditions.

Our analysis also explores how the welfare derived from greenspace might have differed under alternative lockdown rules. We discover that the adoption of more relaxed rules on the use of greenspace during the first period of the lockdown would have delivered increased welfare flow from greenspace of £1.47 billion. A retrospective interpretation of the decision to impose limitations on engagement with greenspace, therefore, would be that the government judged that the health costs associated with the increased risk of infection from adopting less strict rules over that period were in excess of £1.47 billion.

A second counterfactual policy considered the maintenance of the rules limiting engagement with greenspace into the second part of the lockdown. Our analysis reveals that such a policy would have reduced the value flow from greenspace by £1.14 billion. The retrospective interpretation of that figure is that by the time the rules on outdoor recreation were relaxed the government judged that the societal costs of the increased infections that might arise as a consequence, to be less than £1.14 billion.

Several important research questions remain to be answered and the analytical framework developed in this paper stands well placed to address them. As our analysis reveals, behavioural responses to the lockdown differed across the country. In this paper we offer only tentative speculations as to why those differences arose. A more detailed analysis relating the observed changes in outdoor recreation activity to factors including regional differences in the risk of exposure to COVID-19, profiles of occupations, sociodemographics and the local availability and quality of greenspaces might reveal important information as to the key drivers of outdoor recreation behaviour under lockdown. Likewise that detailed exploration of spatial differences in outdoor recreation activity, might help identify those communities that were most seriously disadvantaged by the lockdown restrictions perhaps on account of the lack of availability of high quality local greenspace.

Our analysis also reveals that in the second period of lockdown, use of the outdoors expanded very substantially, far exceeding that expected under normal conditions. The Google Mobility data accessed for the purposes of this analysis provided observations only as far as 7th June 2020. More recent data releases suggest that this increased demand has been maintained even as other areas of everyday life gradually return to normal. That trend has led to speculation that the COVID-19 lockdown has precipitated widespread “re-engagement” with outdoor recreation and is perhaps evidence of a structural shift in preferences for greenspaces (Royal Society for the Protection of Birds 2020). Revisiting the Google Mobility data in a few months’ time and extending the analyses of this paper should help establish the degree of persistence of that shift. If COVID-19 has indeed led citizens of England to discover the delights of the outdoors then perhaps that offers a faint glimmer of positive news in a period so scarred by suffering.

## Footnotes

1 Following academic review, ORVal has been endorsed by the UK government for the valuation of outdoor recreation in the appraisal of public projects and policies (see H.M. Treasury 2018, p. 64).

2 Interestingly, Venter et al. (2020) note that the Google data gives a much lower estimate of the increase in greenspace use in Oslo over lockdown that their own estimate; 19% as compared to 291%.

3 The initial lockdown rules in England restricted citizens to walking to nearby greenspace (HC Deb 24th March 2020). Similar rules were instituted in California (Los Angeles Times, 20th March 2020). In Oslo residents could travel for recreation to any location across the whole municipality (Venter et al. 2020).

4 The regions used by Google are level 2 administrative areas identified by the GADM (release 3.6) datab_a_se (Global Administrative Areas 2020), which aligns very closely to Counties and Unitary Authorities.

5 Regional time series are not always complete. Data points are missing when the numbers engaged in an activity on that day fall below Google’s privacy threshold such that there is insufficient data to ensure anonymity. No further information is provided by Google on this censoring process and in the analyses that follow, we do not attempt to correct for the absence of these data points.

6 The data are taken from the Met Office daily weather series (Met Office 2006a, b) which, at the time of writing, were available up to the 31st May 2020. The weather data are provided for the Met Office’s network of measuring stations across the UK. To arrive at the plots in Fig. 1, temperature and rainfall data were spatially interpolated from the weather station locations to provide daily estimates at each of the 32,844 small area statistical units across England. The England estimates presented in the Figure are population-weighted averages of the temperature and rainfall time series.

7 By June 7th, the last day of the Google data examined in this paper, 11.5 million workers were being paid under UK government schemes that supported the incomes of individuals unable to work as a consequence of the lockdown (Her Majesty's Revenue and Customs [HMRC] 2020).

8 In particular, 45 categories of greenspace were defined and the number of visits to each category recorded in the MENE survey calculated. To ensure representation of less-commonly taken trips, observations were drawn at random from each category with the sampling probability for each category decreasing with the number of observations in each category. Following Manski, and Lerman (1977) we correct for choice-based sampling through reweighting observation in the log-likelihood where the weight for observations in a category are simply the ratio of the population share making that choice to the same share in the sample.

9 Travel costs for driving and walking are calculated from each respondent’s home address to each site through the Ordnance Survey’s detailed road and path network for the UK using state-of-the-art optimal routing algorithms (Dibbelt et al. 2016). Fuel consumption while driving was estimated using formulae provided by the UK Department of Transport (2014) for an average family car and converted to a cost by multiplying by the price of fuel current in the respondent’s region in the month in which they were surveyed. Driving and walking times were converted into costs following guidelines on the valuation of travel time provided by the Department of Transport (2015).

10 Note that while Day and Smith (2017) is a report to the UK government, the ORVal model was developed under expert oversight and subjected to academic review (see research project website: Defra 2018).

11 The socioeconomic segments used in our analysis are the UK Office for National Statistics’ approximated social grade classification which is recorded in the MENE data at 6-category resolution but in the census data at 4-categerory resolution. Accordingly, aggregation adopts the 4-category definition into segments AB, C1, C2 and DE. Dog ownership data is not available at the LSOA level of disaggregation. Rather we take estimates of the proportion of households with a dog at the level of Government Office Region and apply those proportions uniformly to each segment to estimate the population in each segment in each LSOA with and without a dog.

12 Since the baseline spans 2 months we acquire four estimates of these region-visitation quantities; weekdays and weekends in January and February. Given the baseline period comprises 29 days in January and only 6 in February, we reach an estimate of weekday and weekend visitation in the baseline, $\hat{V}_{{gd^{0} }}$, as a weighted sum of the estimates from those 2 months.

13 This is a far from innocuous assumption. That the error term in the numerator of the rhs of Eq. (8) is independent from that in the denominator is similar to our assumption of temporal independence of errors in the estimation of the ORVal model. Less easy to defend is the assumption that errors are independent across regions. Since visits arise from the decisions of individuals choosing between destinations, an event (or conditions) that results in more trips being directed to region $g$ on a particular day will likely result in reduced trips to surrounding regions. We acknowledge that short-coming of our specification, but proceed under the independence assumption to maintain relative simplicity in the calibration procedure.

14 In particular, we calculate the visits from each LSOA to each region assuming Class 1 behaviour, then repeating those calculations for each of the three remaining behavioural classes. Our estimate of visits from LSOA $r$ to region $g$ are calculated as the weighted sum of those four visit estimates where the weights are given by that region’s class-visit shares, $\pi_{h}^{g}$.
